# Substance P participates in periodontitis by upregulating HIF-1α and RANKL/OPG ratio

**DOI:** 10.1186/s12903-020-1017-9

**Published:** 2020-01-30

**Authors:** Kaixian Yan, Qin Lin, Kailiang Tang, Shuang Liu, Yi Du, Xijiao Yu, Shu Li

**Affiliations:** 1 Department of Periodontology, School and Hospital of Stomatology, Shandong University & Shandong Key Laboratory of Oral Tissue Regeneration & Shandong Engineering Laboratory for Oral Tissue Regeneration & Shandong Engineering Laboratory for Dental Materials and Oral Tissue Regeneration, 250012 Jinan, Shandong People’s Republic of China; 2 Department of Endodontics, Jinan Stomatological Hospital, Jinan, Shandong People’s Republic of China

**Keywords:** Substance P, RANKL/OPG, HIF-1α, Osteoclast

## Abstract

**Background:**

Both substance P and hypoxia-inducible factor 1 alpha (HIF-1α) are involved in inflammation and angiogenesis. However, the relationship between substance P and HIF-1α in rat periodontitis is still unknown.

**Methods:**

Ligation-induced rat periodontitis was established to observe the distribution and expression of substance P and HIF-1α by immunohistochemistry. Rat gingival fibroblasts were cultured and stimulated with *Porphyromonas gingivalis* lipopolysaccharide (LPS). Recombinant substance P was applied to elaborate the relationship between substance P and HIF-1α in gingival fibroblasts in vitro. Primary mouse bone marrow-derived macrophages (BMMs) were isolated and cultured to observe the effect of substance P on receptor activator of NF-κB ligand (RANKL)-induced osteoclastogenesis by TRAP staining. Western blotting was used to investigate the expression of HIF-1α, osteoprotegerin (OPG) and RANKL.

**Results:**

Rat experimental periodontitis was successfully established 6 weeks after ligation. Gingival inflammatory infiltration and alveolar bone loss were observed. Positive expression of substance P was found in the infiltrating cells. Higher HIF-1α levels were observed in periodontitis compared to that of normal tissues. Substance P upregulated the level of HIF-1α in gingival fibroblasts with or without 1 μg/ml LPS in vitro (*P < 0.05). Substance P upregulated the expression of HIF-1α in RANKL-stimulated BMMs in vitro. Substance P also increased the RANKL/OPG ratio in gingival fibroblasts (*P < 0.05). Both 10 nM and 50 nM substance P promoted RANKL-induced osteoclast differentiation (*P < 0.05).

**Conclusion:**

Substance P participates in periodontitis by upregulating HIF-1α and the RANKL/OPG ratio.

## Background

Periodontitis is a chronic inflammatory disease with periodontal bone destruction and gingival inflammation [1]. Periodontitis also has an important neurogenic component [2]. Substance P is closely related to neurogenic inflammation and plays a key role in the immune system by regulating the proliferation, migration and activation of immune cells [3]. A number of studies have shown that substance P, which is involved in inflammatory responses [4], exerts certain regulatory functions, such as increasing vascular permeability, affecting vasodilation [5, 6], and inducing angiogenesis [7]. One study found that substance P may have a role in the pathogenesis of periodontal disease [8]. Another study demonstrated that the level of substance P is highest in gingival cervical fluid (GCF) of periodontal destruction sites and that periodontal treatment reduced the amount of substance P [9].

It has been shown that hypoxia and inflammation are closely interrelated [10]. When periodontal microcirculation is locally damaged because of inflammation, there is recruitment of inflammatory cells and activation of multiple O_2_-consuming enzymes in periodontal tissues, which causes obvious hypoxia [11]. In cellular responses to hypoxia, HIF-1α plays a key role [12]. In addition, research has shown that HIF-1α plays a crucial role in physiological and pathophysiological angiogenesis by regulating vascular endothelial growth factor (VEGF) [13, 14].

Both substance P and HIF-1α are closely related to inflammation and angiogenesis. However, the relationship between substance P and HIF-1α in periodontal inflammation is still unknown. Our previous study demonstrated that hypoxia upregulated the expression of RANKL/OPG in human periodontal ligament cells in vitro [15]. In the present study, recombinant substance P with or without LPS was added to rat gingival fibroblasts to observe the expression of HIF-1α, osteoprotegerin (OPG), and receptor activator of NF-kB ligand (RANKL) and the ratio of OPG/RANKL to investigate whether there was a relationships between substance P and HIF-1α in periodontitis.

## Methods

### Animals

Male Wistar rats (220–260 g, Laboratory Animal Center, Shandong University) were maintained on a routine diet to acclimate for 1 week before the experiment. The rats were assigned to two groups at random: a ligation (L) group and a normal (N) group. Protocols of the study met the approval of the Ethics in the Care and Use of Laboratory Animals Committee of the School of Stomatology of Shandong University.

### Rat experimental periodontitis model

Rats in the L group were placed under general anesthesia and underwent an operation to establish the experimental periodontitis model [16]. A 4–0 silk suture and an orthodontic ligature wire were placed around the cervical region of the right first lower molars and then ligated firmly. After 6 weeks, all rats in the two groups were euthanized with a lethal dose (150 mg/kg) of sodium thiopental. The gingiva and alveolar bone tissues were collected and fixed in 4% paraformaldehyde for 48 h. Then, the specimens were dehydrated, cleared and finally embedded in paraffin. Serial sections (5-μm thick) were obtained for hematoxylin-eosin staining (HE) staining and substance P and HIF-1α immunohistochemical staining.

### Cell culture and treatment

Ten Wistar rats (80–100 g) were killed by cervical dislocation. Fresh healthy gingiva was separated and washed three times with phosphate buffered saline (PBS) supplemented with 200 IU/ml penicillin and 200 mg/ml streptomycin (Solarbio, Beijing, China). The gingival tissues were minced by ophthalmic scissors and then digested for 1 hour at 37 °C with a constant temperature shaker in a solution of collagenase type I (3 mg/mL; Solarbio) and dispase (4 mg/mL; Sigma Aldrich, St Louis, USA). After enzymatic digestion, the filtered single-cell suspension was maintained in α-minimal essential medium (α-MEM; HyClone, Logan, USA) containing 20% fetal bovine serum (FBS; Biological Industries, Kibbutz, Israel), 100 IU/ml penicillin and 100 mg/ml streptomycin at 37 °C in an incubator with a 95% O_2_–5% CO_2_ atmosphere. After reaching confluence, the cells were detached with 0.25% Trypsin-EDTA solution (Solarbio) and subcultured in α-MEM with 10% FBS. The medium was changed every 48 h. Cells between the fourth and sixth passages were used for subsequent experiments.

Primary mouse bone marrow-derived macrophages (BMMs) were isolated from the femurs and tibias of 10 C57/BL6 male mice (3 weeks old) after cervical dislocation and were cultured in complete α-MEM containing 10% FBS and 30 ng/ml macrophage colony stimulating factor (M-CSF) at 37 °C in an incubator with a 95% O_2_–5% CO_2_ atmosphere. We added 50 ng/ml RANKL for 4 days to induce BMMs to differentiate into osteoclasts. To observe the effect of substance P on osteoclastogenesis, we added 10 nM substance P (Sigma Aldrich) with or without 1 μg/ml LPS (Invivo Gen, San Diego, USA) to the culture medium (RANKL+ 10 nM SP group and RANKL+ 50 nM SP group, RANKL only group as control) and then stained for TRAP.

### TRAP staining

The cells were fixed with 4% paraformaldehyde and then stained for TRAP using a commercially available kit (Joy Tech Bio. Co., Hangzhou, China). Osteoclasts were identified as TRAP-positive multinucleated cells containing three or more nuclei.

### Immunohistochemical staining

After deparaffinization using xylene and hydration in gradient ethanols, the tissue sections were treated with 3% H_2_O_2_ for 10 min at room temperature to inhibit endogenous peroxidase activities and then incubated with primary antibodies against substance P (diluted 1:200, Abcam, Cambridge, UK) and HIF-1α (diluted 1:200, Abcam) overnight at 4 °C. The method was the same as our previously described research. After washing with 0.01 M PBS, the sections were incubated with polymer auxiliary agent for 15 min at 37 °C, washed with 0.01 M PBS 5 min × 3 times, and then incubated with Poly-HRP secondary antibody goat anti-mouse/goat anti-rabbit IgG (ZSBio, Beijing, China) for 15 min at 37 °C. After three washes in 0.01 M PBS for 3 min each, the sections were visualized with 3,3-diaminobenzidine tetrahydrochloride (ZSBio) as recommended by the manufacturer. The negative control used 0.01 M PBS instead of antibodies. The sections were examined and photographed with a light microscope (OLYMPUS CX-71, Japan).

### Western blot analysis

RIPA lysis buffer (Solarbio) was used to extract the total proteins. The protein concentrations were measured by using a bicinchoninic acid (BCA) assay kit (Solarbio) according to the manufacturer’s instructions. Equal loading quantities of proteins were separated by 10% SDS-PAGE and electroblotted to polyvinylidene difluoride (PVDF) membranes (Millipore, Billerica, USA). The membranes were blocked with 5% nonfat milk dissolved in TBST at room temperature for 1 h and then incubated overnight at 4 °C with primary antibodies against HIF-1α (diluted 1:500, Abcam), TNF-α (diluted 1:500, Abcam), OPG (diluted 1:500; Bioss, Beijing, China) and RANKL (diluted 1:500; Bioss). After washing with TBST, the membranes were incubated with secondary horseradish peroxidase (HRP)-linked goat-anti rabbit IgG antibody (diluted 1:10000, CWBiotech, Beijing, China) at room temperature for 1 h. The blots were visualized by using an ECL kit (Millipore).

### Statistical analysis

All data are expressed as the mean ± SD. Unpaired Student’s t-tests were conducted with GraphPad Prism 5 software. The results for multiple group comparisons were analyzed using one-way analysis of variance (ANOVA) followed by a Newman–Keuls post hoc test. A value of *P* < 0.05 was considered statistically significant.

## Results

### Rat experimental periodontitis model

**Fig. 1:**
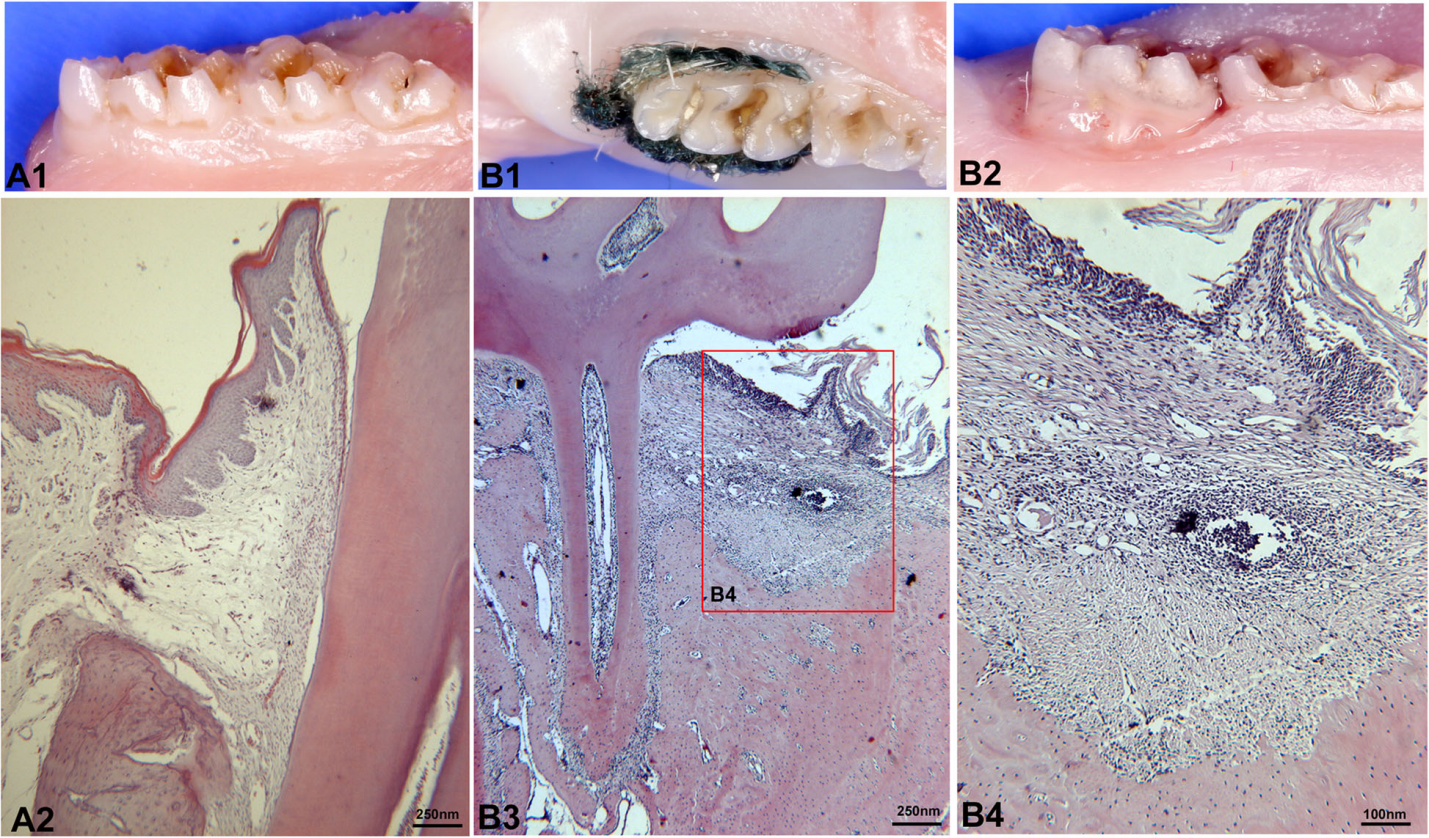
Rat experimental periodontitis model. Healthy gingival tissue attached to teeth cervical region in Normal group (A1). Six weeks after ligation, the ligatures still firmly stayed on cervical region (B1). Obvious gingival recession of the first molars was observed at 6 weeks after ligation (B2). Compared with normal periodontal tissues (A2), gingival inflammatory infiltration and obvious alveolar bone loss were observed by hematoxylin-eosin staining (B3, B4)

Obvious gingival recession of the first molars was observed at 6 weeks after ligation (Fig. 1B2). Gingival inflammatory infiltration and obvious alveolar bone loss were observed by hematoxylin-eosin staining (Fig. 1B3, B4) compared to that of normal periodontal tissues (Fig. 1A2).

### Expression of HIF-1α and substance P in rat ligation-induced experimental periodontitis

**Fig. 2:**
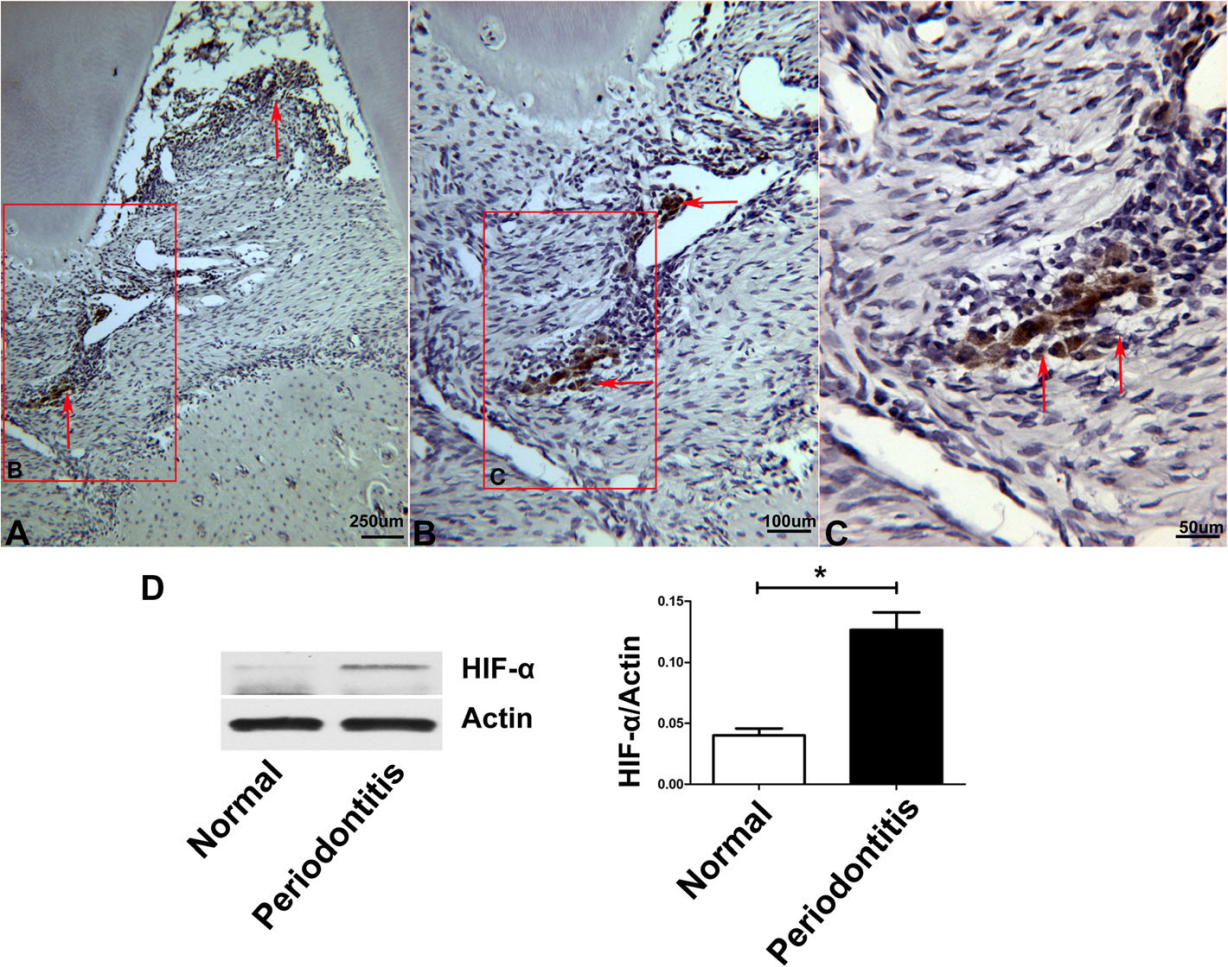
Expressions of HIF-1α in rat ligation-induced experimental periodontitis. Immunohistochemistry staining showed positive expression of HIF-1α in the region where infiltrating inflammatory cells were localized (**a**, **b**, **c**). Compared with normal gingiva, the gingiva in periodontitis expressed higher HIF-1α according to western blotting (*P < 0.05) (**d**)

**Fig. 3:**
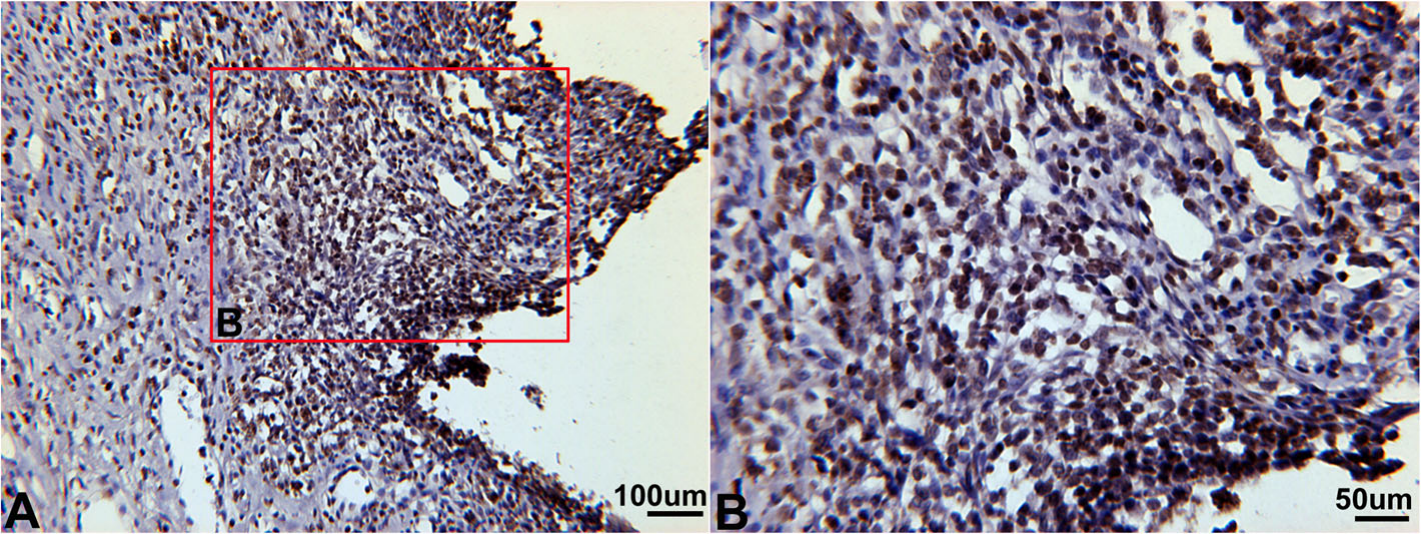
Expression of substance P in rat ligation-induced experimental periodontitis. The positive staining of substance P was localized in inflammatory infiltrating cells in the gingiva of rat experimental periodontitis (**a**). It was more clear in local enlarged vision (**b**)

Immunohistochemistry staining showed positive expression of HIF-1α in the region where infiltrating inflammatory cells were localized (Fig. 2a, b, c). Western blotting showed that the gingiva in periodontitis expressed higher HIF-1α compared with that of normal gingiva (*P < 0.05) (Fig. 2d). In addition, we observed that the positive staining of substance P was the same as that of HIF-1α (Fig. 3).

### Substance P upregulated the level of HIF-1α in gingival fibroblasts

**Fig. 4:**
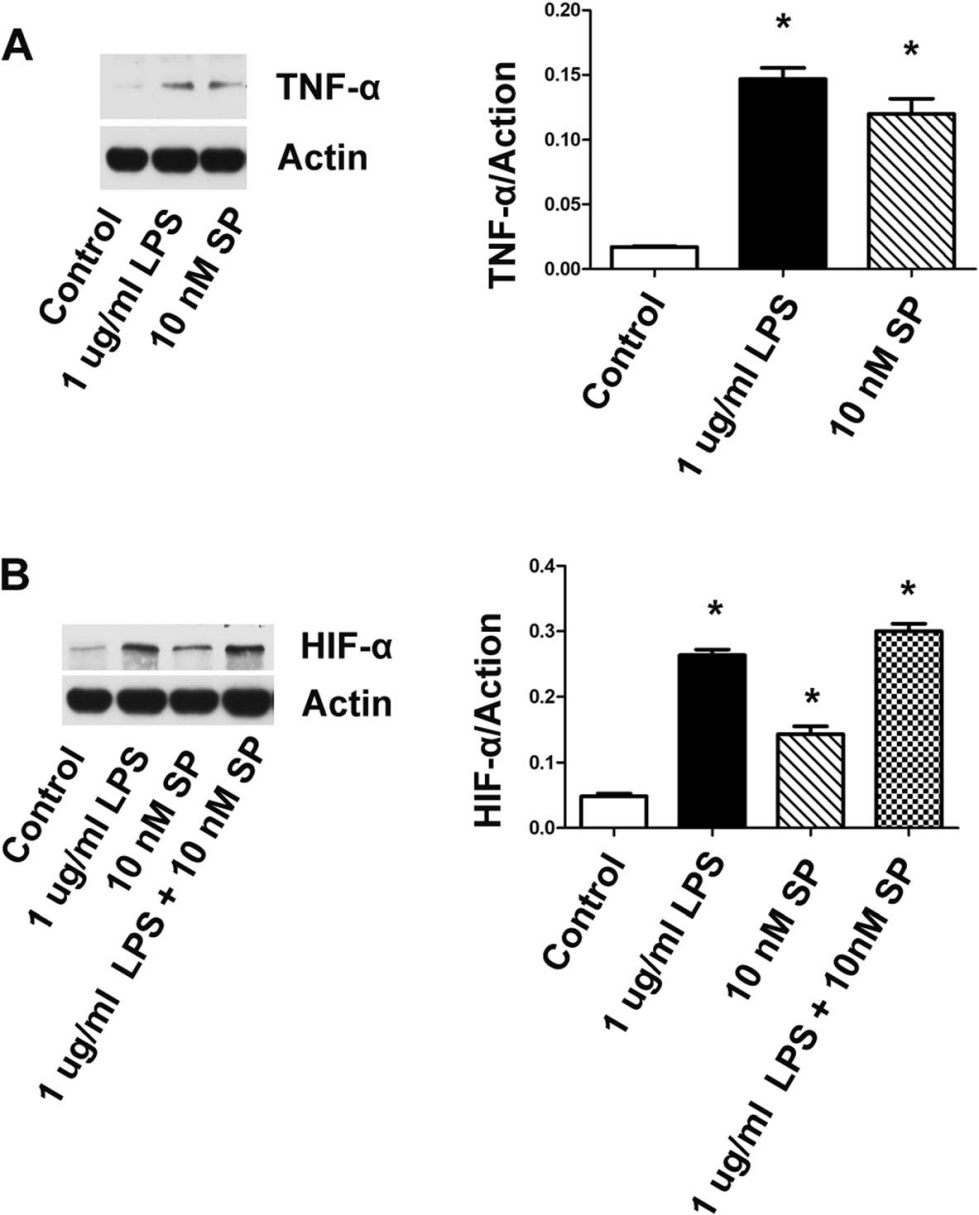
Substance P upregulated the level of HIF-1α in gingival fibroblasts. After 24 h, both 1 μg/ml LPS and 10 nM substance P obviously induced TNF-α expression (*P < 0.05) (**a**). The level of HIF-1α was upregulated by applying of 10 nM SP with or without 1 μg/ml LPS in gingival fibroblasts by western blotting (*P < 0.05) (**b**)

After 24 h, both 1 μg/ml LPS and 10 nM substance P obviously induced TNF-α expression (*P < 0.05) (Fig. 4a). The results showed that 10 nM substance P with or without 1 μg/ml LPS upregulated the level of HIF-1α in gingival fibroblasts (*P < 0.05) (Fig. 4b), while 10 nM substance P with 1 μg/ml LPS induced the highest upregulation of HIF-1α expression.

### Substance P promoted RANKL-induced osteoclast differentiation in vitro

**Fig. 5:**
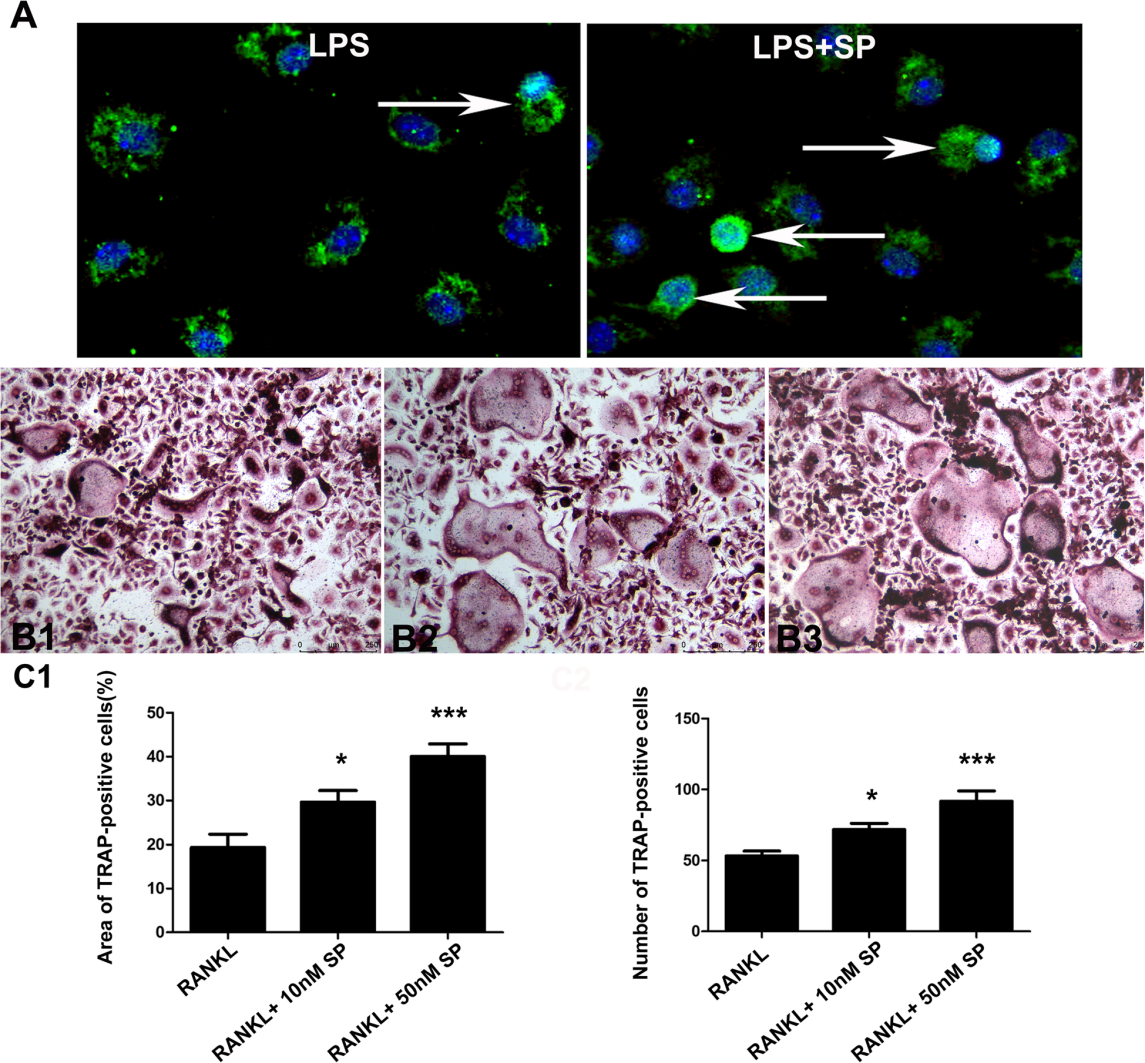
Substance P promoted RANKL-induced osteoclast differentiation in vitro. The expression of HIF-1α was observed in BMMs stimulated with 1 μg/ml LPS with or without 10 nM substance P. Increased expression was found in the LPS + SP group (**a**). Compared with the RANKL group (B1), more TRAP positive osteoclasts were detected in the RANKL+ 10 nM SP (B2) and RANKL+ 50 nM SP groups (B3). Both 10 nM and 50 nM substance P upregulated RANKL-induced osteoclast differentiation (*P < 0.05) (C1)

The expression of HIF-1α was observed in BMMs stimulated with 1 μg/ml LPS with or without 10 nM substance P. Increased expression was found in the LPS + SP group (Fig. 5a). In the RANKL+ 10 nM SP (Fig. 5B2) and RANKL+ 50 nM SP groups (Fig. 5B3), more TRAP-positive osteoclasts were detected than in the RANKL group (Fig. 5B1). Both 10 nM and 50 nM substance P upregulated RANKL-induced osteoclast differentiation (Fig. 5c).

### Substance P upregulated the RANKL/OPG ratio in gingival fibroblasts

**Fig. 6:**
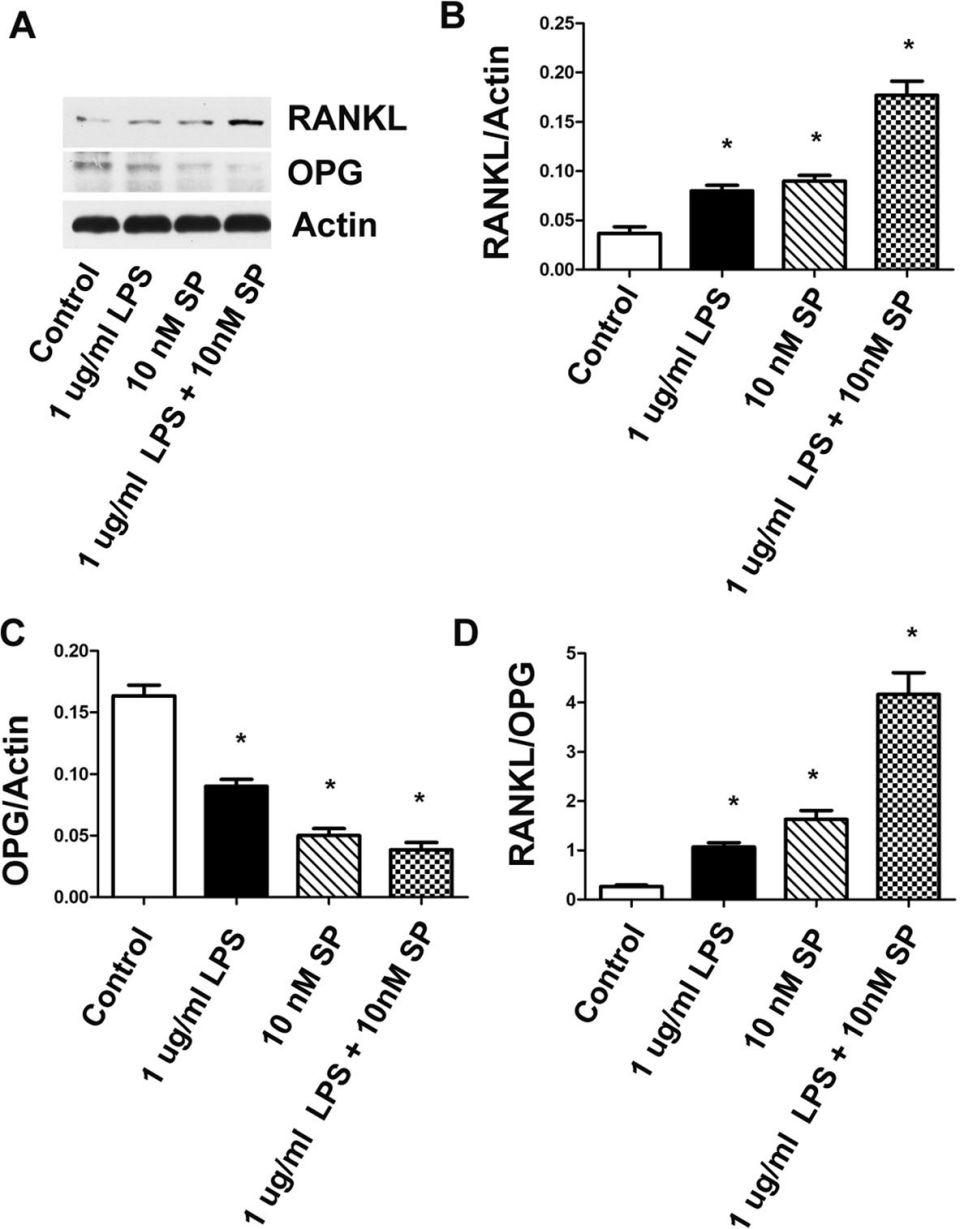
Substance P upregulated RANKL/OPG ratio in gingival fibroblasts tested by western blotting. Substance P upregulated RANKL protein expression and reduced OPG protein expression in gingival fibroblasts with or without 1 μg/ml LPS (*P < 0.05) (**a**, **b**, **c**). The RANKL/OPG ratio was markedly increased in the LPS + SP group, compared to the LPS only group (*P < 0.05) (**d**)

The ratio of RANKL/OPG in gingival fibroblasts was tested by western blotting. Substance P upregulated RANKL protein expression and reduced OPG protein expression in gingival fibroblasts with or without 1 μg/ml LPS (*P < 0.05) (Fig. 6a, b, c). The RANKL/OPG ratio was markedly increased in the LPS + SP group compared to that of the LPS only group (*P < 0.05) (Fig. 6d).

## Discussion

In this study, increased expression of HIF-1α and substance P was observed in rat ligation-induced experimental periodontitis, which revealed that both factors participate in periodontitis.

Substance P is induced by cytokines and LPS, is characterized as a proinflammatory neurotransmitter and plays a key role in inflammation [2]. Increased substance P is also detected in gingival tissues and GCF of periodontitis [9], which is correlated with periodontal inflammation [17]. In this study, 10 nM substance P obviously upregulated the level of TNF-α in gingival fibroblasts. TNF-α, a proinflammatory cytokine, is able to further induce tissue destruction and bone resorption [18].

Substance P inhibits osteoblast differentiation and may be related to bone metabolism in periodontal diseases under conditions of stress [17, 19]. Osteoclasts express the substance P receptor, which binds to substance P and then induces osteoclastogenesis. Constant production of a certain quantity of substance P results in bone resorption [20, 21]. Our study showed that substance P upregulated osteoclast differentiation induced by RANKL. In addition, substance P also markedly increased the RANKL/OPG ratio in LPS-simulated gingival fibroblasts. An increased RANKL/OPG ratio, which denotes the occurrence of osteoclastogenesis, promotes bone resorption [22], which was consistent with the result of Lee et al. [23]. Substance P might participate periodontitis by altering the RANKL/OPG ratio.

In the regulation of oxygen homeostasis, it has been fully testified that HIF-1α is an essential transcriptional regulator [24]. Under normal oxygen conditions, HIF-1α is hardly undetectable. When cells are in hypoxic conditions, the level of the HIF-1α protein obviously increases [25]. Periodontal tissues have also been shown to be relatively hypoxic and ischemic in periodontitis [1]. The activation of HIF-1 is linked to altered immunity and inflammation [26, 27]. In this study, the addition of 10 nM substance P obviously upregulated the level of HIF-1α in gingival fibroblasts. Our previous study showed that hypoxia changed the metabolic pathway of human periodontal ligament (PDL) fibroblasts by upregulating the expression of HIF-1α, VEGF and other relevant growth factors [15]. Another study showed that HIF-1α seemed to be involved in the induction, progression, and persistence of periodontitis [1]. Inflammatory cytokines such as LPS are able to induce the expression of HIF-1α in an NF-κB-dependent manner under normoxic conditions in human PDL fibroblasts [1]. Our study showed that substance P upregulated the expression of HIF-1α in LPS-stimulated BMMs. Substance P might participate in periodontitis by upregulating HIF-1α. However, Hirai reported that activation of hypoxia-inducible factor 1 attenuates periapical inflammation and bone loss [28]**.** That study established periapical lesions in mice, while our study established experimental periodontitis in rats, and the dominant microorganisms must be distinct from each other because of different operative methods and oxygen conditions. In vitro experiments demonstrated that HIF-1α suppressed the inflammatory response in endodontic pathogen-stimulated macrophages via downregulation of NF-κB promoter activity. In our study, we used LPS as the stimulator, which is the main pathogen in periodontitis. The function and mechanisms of HIF-1α in inflammation needs further analysis under specific conditions such as cell types, animal species, and microenvironment. On the one hand, HIF-1α allows cells adapt to a reduced-oxygen environment, as mentioned above. Proper amounts of HIF-1α contribute to inhibiting inflammation [29]. On the other hand, HIF-1α enhances IL-1 production and promotes inflammatory responses [30]. The application of HIF-1α also increases the activity of NF-κB [31, 32].

More attention should be paid to the roles of HIF-1α in periodontitis and further study is needed. Applying a HIF-1α antagonist may be helpful for interpreting the relationship between HIF-1α and substance P in periodontitis and observing the role of substance P in osteoclast differentiation. We will carry out the relevant experiments in future studies to investigate the mechanisms of substance P and HIF-1α in periodontitis.

## Conclusion

Substance P participates in periodontitis by upregulating HIF-1α and the RANKL/OPG ratio. The roles of HIF-1α in periodontitis should be further studied.

## Data Availability

The data and materials used in the present study are available from the corresponding authors on reasonable request.

## Abbreviations

BCA: Bicinchoninic acid
BMMs: Bone marrow macrophages
BMMs: Bone marrow-derived macrophages
FBS: Fetal bovine serum
GCF: Gingival cervical fluid
HE: Hematoxylin-eosin staining
HIF-1α: Hypoxia-inducible factor-1 alpha
HRP: Horseradish peroxidase
L: Ligation
LPS: Lipopolysaccharide
M-CSF: Macrophage Colony Stimulating Factor
N: Normal
OPG: Osteoprotegerin
PBS: Phosphate buffer
PVDF: Polyvinylidene difluoride
RANKL: NF-kB ligand
SP: Substance P
TRAP: Tartrate-resistant acid phosphatase
α-MEM: α-Minimal essential medium
